# Prevalence of anemia before and after initiation of highly active antiretroviral therapy among HIV positive patients in Northwest Ethiopia: a retrospective study

**DOI:** 10.1186/1756-0500-7-745

**Published:** 2014-10-22

**Authors:** Zelalem Tesfaye, Bamlaku Enawgaw

**Affiliations:** Department of Medical laboratory Science, College of Medicine and Health Sciences, University of Gondar, Gondar, Ethiopia; Department of Hematology & Immunohematology, School of Biomedical and Laboratory Sciences, College of Medicine and Health Sciences, University of Gondar, P.O. Box 196, Gondar, Ethiopia

**Keywords:** HIV, Anemia, HAART

## Abstract

**Background:**

Human immunodeficiency virus (HIV) associated anemia is common and it is a challenge for prognosis of HIV positive patients. It is stated in different literature that the prevalence of anemia due to HIV before highly active antiretroviral therapy (HAART) initiation is more prevalent than after HAART initiation. Thus this study aimed to assess anemia prevalence before and after initiation of HAART among HIV patients attending university of Gondar hospital from 2008 – 2013.

**Methods:**

A retrospective study was conducted by collecting data from antiretroviral clinic of University of Gondar Hospital from January 1, 2008 to December 30, 2013. Data was collected by using data collection sheet which contains age, sex, regimen type, hematological parameters and CD4 cell count. Data were analyzed using SPSS version 20. In order to compare means paired t-test was used. P- Value <0.05 was considered as significant.

**Results:**

Prevalence of anemia before and after HAART initiation was 21.2% and 11.5% respectively. There is a significance difference in CD4 cell count, hemoglobin and hematocrit values on patients before and after HAART initiation (P <0.001). Opportunistic infection and CD4 cell count were associated with prevalence of anemia before HAART initiation.

**Conclusion:**

There was a decline in the prevalence of anemia and increment of mean CD4 cell count among HIV infected patients after HAART initiation. HIV patients are recommended checking up their CD4 cell counts regularly and starting HAART when it is appropriate with regular follow-up.

## Background

Anemia is the most common hematological abnormality in HIV positive patients. It has been shown to be the risk factor for early death in patients with AIDS. Anemia is not only associated with decreased quality of life but it is also independently associated with decreased survival with HIV [1]. It is developed in close to 95% of HIV infected patients before the initiation of HAART, and still found in up to 46% of patients in the HAART [2, 3].

Previous studies have found that the incidence of anemia increases with progression of HIV infection. Several other etiologic factors may also be involved in the development of HIV-associated anemia, including micronutrient deficiencies, immunological myelosuppression, impaired erythropoietin production and blood loss from intestinal opportunistic disease [4].

Anemia is a condition in which there is a decrease in the red blood cell count, hemoglobin and/or hematocrit values as compared to normal reference range for age, sex, race and altitude which decreases oxygen-carrying capacity of red blood cells to tissues [5]. It is defined as a hemoglobin level <12 g/dl for adult female and <13 g/dl for adult male, in accordance with WHO guidelines and classified as mild (11–11.9 g/dl for women and 11–12.9 g/dl for men), moderate (8–10.9 g/dl) and severe (<8 g/dl) [6].

Anemia is observed in both antiretroviral therapy treated and untreated individuals, but severity varies due to the immune status or CD4 cell levels and also the prevalence increases as the disease advanced and left untreated [7]. If an individual used antiretroviral treatment effectively, there will be an increased level of CD4 cells and hemoglobin concentration, which in turn decrease the occurrence of anemia [8–14]. Although HIV infected patients on HAART showed lower prevalence of anemia, some reports indicated that AZT can induces anaemia [15].

Most studies described the prevalence of anemia in HIV infected individuals before and after initiation of highly active antiretroviral therapy (HAART), however few studies were conducted in Ethiopia on the assessment of anemia among HIV positive patients [11, 12, 16]. Therefore, this study gave information about the anemia before and after initiation of antiretroviral therapy among patients who attend at ART clinic of Gondar University Hospital.

## Methods

### Study setting and study population

This retrospective study was conducted in the ART clinic of the University of Gondar Hospital which is found in Northwest of Ethiopia. Patients’ data with full information of sex, age, regimen type, hematological and immunological parameters, WHO clinical stage, type of regimen, opportunistic infections and cotrimoxazole were collected from follow up charts and registration books of patients who started HAART at GUH ART clinic from January 1, 2008 to December 30, 2013. Hematological and immunological parameters were collected at baseline when the HIV positive patients adhere to ART and after six month of HAART initiation. The quality of data were controlled at different levels for completeness and consistency. Whenever an error was found at any level, it was corrected by rechecking from the registration book.

### Statistical analysis

The data were cleaned, edited, checked for completeness and entered into SPSS version 20 for analysis. Descriptive statistics was used to get a clear picture of dependent and independent variables. In order to compare means paired t-test was used and also chi square was computed to determine association between dependent and independent variables. P- Value <0.05 was considered as significant.

### Ethical consideration

The study was conducted after ethical letters obtained from School of Biomedical and Laboratory Science Ethical Committee. Then permission was taken from hospital higher management. After permission was obtained, data were collected from ART clinic. To keep confidentiality codes were used and unauthorized person didn’t have access to the data.

## Results

### General characteristics of study participants

A total of 349 HIV positive patients, 218 (62.5%) females and 131 (37.5%) males, were involved in this study. The overall mean age was 34.6 ± 8.5 years, within the range of 15 – 60 years old. Majority of the patients [154 (44.1%)] were within 26 – 35 years of age. About 226 (64.8%) of participants were under WHO clinical stage I & II, 87.7% of them were taking cotrimoxazole prophylaxis therapy and about 4.3% of them were TB co-infected (Table 1).

**Table 1:** **Socio demographic and clinical characteristics of HIV positive patients attending University of Gondar hospital, North West Ethiopia from 2008-2013**

Variables		Frequency	Percentage
**Sex**	Male	131	37.5
	Female	218	62.5
**Age**	<25 years	46	13.2
	26-35 years	154	44.1
	36-45 years	114	32.7
	>46 years	35	10.0
**Regimen type**	AZT based	215	61.6
	Non AZT based	134	38.4
**Cotrimoxazole**	Yes	306	87.7
	NO	43	12.3
**Opportunistic infections (OPIs)**	Yes	15	4.3
	No	334	95.7
**WHO stage**	WHO stage I & II	226	64.8
	WHO stage III & IV	123	35.2

### Laboratory profile of HIV positive patients

The mean WBC count, hemoglobin, platelet and CD4 cell count before HAART initiation were 5.62 ± 2.13 cells/μl, 13.65 ± 2.14 g/dl, 279.33 ± 101 cells/μl and 251.72 ± 164.15 cells/μl and after HAART initiation were 5.58 ± 2 cells/μl, 14.04 ± 1.75 g/dl, 289.15 ± 99.43cells/μl and 295.85 ± 155 cells/μl respectively. Patients after HAART initiation have high hemoglobin level, hematocrit and CD4 cell count when compared to before HAART initiation patients (P <0.001) (Table 2).

**Table 2:** **Hematological parameters of HIV positive patients before and after initiation of HAART at University of Gondar Hospital Northwest Ethiopia from 2008 – 2013**

Parameters	Before HAART initiation	After HAART initiation	95% CI	P – Value
	Mean ± SD	Mean ± SD		
**WBC**	5.62 ± 2.13	5.58 ± 2	-0.175 – 0.253	0.722
**Lymphocyte**	1.95 ± 1	1.93 ± 0.86	-0.101 – 0.127	0.822
**Mixed**	0.63 ± 0.33	0.6 ± 0.27	-0.05 – 0.062	0.096
**Granulocyte**	3.04 ± 1.61	3.05 ± 1.45	-0.159 – 0.151	0.958
**Hemoglobin**	13.65 ± 2.14	14.04 ± 1.75	-0.174 – (-)3.55	0.000*
**Hematocrit**	40.4 ± 6.34	42.15 ± 5.67	-1.05 – (-) 4.88	0.000*
**Platelet**	279.33 ± 101	289.15 ± 99.43	-20.789 – 1.156	0.079
**CD4 cells//μl**	251.72 ± 164.15	295.85 ± 155	-30.61 – (-)6.42	0.000*

**Note:** Level of significance (P Value) is determined based on paired sample T test.

Numbers with atrix (*) indicates significance.

### Prevalence of anemia among HIV positive patients

**Figure 1:**
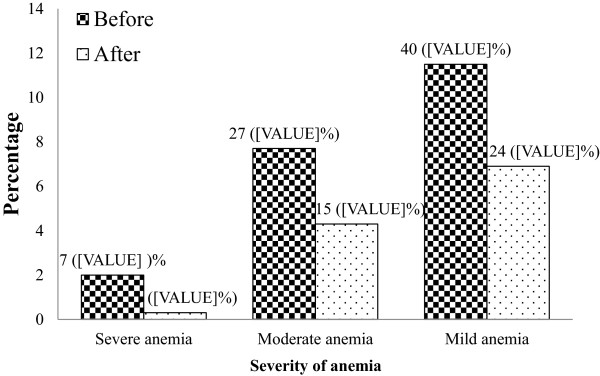
**Severity of anemia among HIV/AIDS patients at the University of Gondar Hospital Northwest Ethiopia.** The figure indicates that the severity of anemia was higher before HAART initiation among HIV positive patients.

The prevalence of anemia in HIV patients was 21.2% (74/114) before HAART initiation and 11.5% (40/114) after HAART initiation. About 40 (11.5%) had mild anemia, 27 (7.7%) had moderate anemia, and 7 (2%) had severe anemia before HAART initiation. However, the prevalence of anemia after HAART initiation was significantly decreased by 9.7% (Figure 1).

### Risk factors of anemia in HIV infected patients

From anemic patients before HAART initiation, about 19.8% (26/131) were males and 22% (48/118) were females. Similarly 30.4% (14/46) patients at the age of <25 years old and 35.2% (50/142) of patients with CD4 cell count <200 cells/μl were developed anemia. There was a significant association between anemia with CD4 cell count and TB before HAART initiation. HIV patients with CD4 cell count <200 cells/μl before HAART initiation had higher prevalence of anemia (35.2%, P =0.001). Similarly about 53.3% (P =0.002) patients with TB had developed anemia before HAART initiation. However, there was no association of anemia with sex, age, and WHO clinical stage (Table 3).

**Table 3:** **Anemia and its associated factor before HAART initiation in HIV patients attending University of Gondar hospital, North West Ethiopia from 2008-2013**

Variables		Anemic	Non-anemic	X ^2^	P value
**Sex**	Male	26 (19.8%)	105 (80.2%)	0.23	0.63
	Female	48 (22%)	170 (78%)		
**Age**	<25 years	14(30.4%)	32(69.6%)	3.44	0.33
	26-35 years	32(20.8%)	122(79.2%)		
	36-45 years	23(20.2%)	91(79.8%)		
	>46 years	5(14.3%)	30(85.7%)		
**Opportunistic infection**	Yes	8(53.3%)	7(46.7%)	9.68	**0.002**
	No	66(19.8%)	268(80.2%)		
**WHO stage**	WHO stage I & II	42(30.1%)	184(169.7%)	2.61	0.455
	WHO stage III & IV	32(53.7%)	91(145.7%)		
**CD4 cells//μl**	<200	50(35.2%)	92(64.8%)	31.25	**0.000**
	200-350	20(15.5%)	109(84.5%)		
	>350	4(5.1%)	74(94.9%)		

**Note:** Level of significance (P value) derived from chi square test, Numeric numbers in bold indicates statistical significant.

From anemic patients after HAART initiation, about 7.6% (10/131) were males and 13.8% (30/118) were females. Similarly 12.3% (14/146) patients at the age of 36–45 years old and 18% (18/100) patients with CD4 cell count <200 cells/μl were developed anemia. Although, HIV patients with CD4 cell count <200 cells/μl had higher prevalence of anemia (18%) after HAART initiation, there was no significance association between anemia and CD4 cell count (P =0.051). Similarly sex, age regimen type and WHO clinical stage had no association with anemia (Table 4).

**Table 4:** **Anemia and its associated factor after HAART initiation in HIV patients at University of Gondar hospital, North West Ethiopia from 2008-2013**

Variables		Anemic	Non-anemic	X ^2^	P value
**Sex**	Male	10(7.6%)	121(92.4%)	3.028	0.082
	Female	30(13.8%)	188(86.2)		
**Age**	<25 years	6(13%)	40(87%)	1.336	0.721
	26-35 years	18(11.7%)	136(88.3%)		
	36-45 years	14(12.3%)	100(87.7%)		
	>46 years	2(5.7%)	33(94.3%)		
**Regimen type**	AZT based	24(11.2%)	191(88.8%)	0.049	0.825
	Non AZT based	16(11.9%)	118(88.1%)		
**Cotrimoxazole**	Yes	35(11.4%)	271(88.6%)	0.001	0.971
	No	5(11.6%)	38(88.4%)		
**CD4 cells//μl**	<200	18(18%)	82(82%)	5.953	0.051
	200-350	12(8.5%)	130(91.5%)		
	>350	10(9.3%)	97(90.7%)		

## Discussion

In this study prevalence of anemia was 21.2% and 11.5% before HAART initiation and after HAART initiation respectively. This indicates that prevalence of anemia was higher in untreated patients. This is consistent with study conducted in Minillik II hospital ART clinic, Addis Ababa Ethiopia 200 (52.6%) and (37.3%) [12], Hawassa from 2007–2011 (86.5%) and (80.5%) before HAART initiation and after HAART initiation [13]. This is due to the fact that HIV disease progression is directly related to the prevalence and the severity of anemia. Anemia has been shown to be statically significant predictor of progression to the AIDS and is independently associated with an increased risk of death in patient with HIV [6]. Treatment with HAART suppresses viral replication and increases CD4 cell count [17]. This in turn increases immunity and decreases the effect of HIV on hematopoietic stem cells and different opportunistic infections [7].

Prevalence of anemia before HAART initiation (21.2%) in this study was lower than study done in different countries, such as Germany from 2001–2002 (61%) [8], India in 2008 (84.6%) [10], Baltimore Maryland (40%) [9], Tanzania (77.4% [16], Minillik II hospital, Addis Ababa in 2000 (52.6%) [12] and Hawassa from 2007 – 2011(86.5%) [13]. This discrepancy is due to the difference in study population, socio demographic characteristics and study design methods.

Prevalence of anemia after HAART initiation (11.5%) was lower study done in in studies from Minillik II hospital, Addis Ababa 200 (37.3%) [12] and Hawassa from 2007–2011 (80.5%) [13]. This is due to the difference in the awareness of the patients about HAART was increased and use as recommendation and the time of the study done.

The prevalence of anemia observed across sex groups before HAART initiation was 19.8% (26/131) for males and 22% (48/218) for females similarly after HAART initiation 7.6% (10/131) and 13.8% (30/218) were male and female patients were anemic respectively. This was slightly lower than a report from Minillik II hospital ART clinic, Addis Ababa Ethiopia, 70.25% vs. 29.75% in males and females before HAART initiation and 69.23% vs. 30.77% in males and females after HAART initiation respectively [12]. Prevalence of anemia was decreased after HAART initiation from 19.8% to 7.6% in males and from 22% to 13.8% in females.

In this study the minimum and maximum CD4 cell count before HAART initiation was 1 cell/μl and 795 cells/μl and after HAART initiation were 14 cells/μl and 969 cells/μl. Patients with CD4 cell count <200 cells/μl before HAART initiation was 142 (40.7%) while after HAART initiation was 100 (28.7%). The risk of having CD4 cell count <200 cells/μl was increased before HAART initiation, because before HAART initiation patients have less immunity towards the HIV and the virus attacks CD4 cells easily.

## Conclusion

There was a decline in the prevalence of anemia and increment of mean CD4+ T cell count among HIV infected patients after HAART initiation. Before HAART initiation, HIV patients have significantly higher prevalence of anemia than those after HAART initiation. The prevalence of anemia was significantly higher in patients with CD4 cell count <200 cell/μl. There was a significant association between prevalence of anemia with CD4 cell count and opportunistic infections before HAART initiation. But there was no association of prevalence of anemia with sex, age, regimen type and WHO clinical stage. Based on the present finding, HIV patients are recommended to check up their CD4 cell counts regularly and start HAART when it is appropriate in order to decrease the prevalence of anemia.

## Abbreviations

AIDS: Acquired immunodeficiency syndrome
ART: Antiretroviral treatment
AZT: Azidothymidine
CD4: Cluster of differentiation 4
CD8: Cluster of differentiation 8
D4T: Stavudine
GUH: Gondar university hospital
HAART: Highly active antiretroviral therapy
HCT: Hematocrit
Hgb: Hemoglobin
HIV: Human immunodeficiency virus
IDUs: Injection drug users
MCH: Mean cell hemoglobin
MCHC: Mean cell hemoglobin concentration
MCV: Mean cell volume
PLT: Platelet
RBC: Red blood cell
RDW: Red cell distribution width
WBC: White blood cell
WHO: World Health Organization.
